# Melatonin receptor depletion suppressed hCG-induced testosterone expression in mouse Leydig cells

**DOI:** 10.1186/s11658-019-0147-z

**Published:** 2019-03-12

**Authors:** Yuan Gao, Xiaochun Wu, Shuqin Zhao, Yujun Zhang, Hailong Ma, Zhen Yang, Wanghao Yang, Chen Zhao, Li Wang, Quanwei Zhang

**Affiliations:** 1 College of Life Science and Technology, Gansu Agricultural University, Lanzhou, 730070 Gansu China; 2 College of Veterinary Medicine, Gansu Agricultural University, Lanzhou, Gansu China

**Keywords:** Melatonin receptor, Testosterone, ER stress, Apoptosis

## Abstract

Melatonin receptors MT1 and MT2 (genes officially named MTNR1A and MTNR1B, respectively) play crucial roles in melatonin-mediated regulation of circadian rhythms, the immune system, and control of reproduction in seasonally breeding animals. In this study, immunolocalization assay showed that MT1 and MT2 are highly expressed in Leydig cell membrane. To understand the biological function of melatonin receptors in hCG-induced testosterone synthesis, we generated melatonin receptor knockdown cells using specific siRNA and performed testosterone detection after hCG treatment. We found that knockdown of melatonin receptors, especially MTNR1A, led to an obvious decrease (> 60%) of testosterone level. Our further study revealed that knockdown of melatonin receptors repressed expression, at both the mRNA level and the protein level, of key steroidogenic genes, such as p450scc, p450c17 and StAR, which are essential for testosterone synthesis. hCG triggered endoplasmic reticulum (ER) stress to regulate steroidogenic genes’ expression and apoptosis. To further investigate the potential roles of melatonin receptors in hCG-induced regulation of ER stress and apoptosis, we examined expression of some crucial ER stress markers, including Grp78, Chop, ATF4, Xbp1, and IRE1. We found that inhibition of melatonin receptors increased hCG-induced expression of Grp78, Chop and ATF4, but not Xbp1 and IRE1, suggesting that hCG may modulate IRE1 signaling pathways in a melatonin receptor-dependent manner. In addition, our further data showed that knockdown of MTNR1A and MTNR1B promoted hCG-induced expression of apoptosis markers, including p53, caspase-3 and Bcl-2. These results suggested that the melatonin receptors MTNR1A and MTNR1B are essential to repress hCG-induced ER stress and cell apoptosis. Our studies demonstrated that the mammalian melatonin receptors MT1 and MT2 are involved in testosterone synthesis via mediating multiple cell pathways.

## Introduction

Melatonin (N-acetyl-5-methoxytryptamine), a neuro-hormone that is mainly secreted from the pineal gland in all mammals, influences various physiological activities such as neuroendocrine function, regulation of seasonal reproduction, sexual maturation, immunoregulation, thermoregulation, some aspects of aging and strong antioxidant activity [1–5].

Melatonin’s physiological actions are mainly mediated by two types of melatonin receptors, MT1/Mel1a and MT2/ Mel1b (genes officially named MTNR1A and MTNR1B, respectively). Both the MT1 and MT2 receptors are classified as class A rhodopsin type G-protein coupled receptors (GPCRs) with typically seven transmembrane domains, connected to each other by three extracellular regions and three intracellular loops [6, 7]. The two receptors have 60% homology and have been reported in rats, mice, and humans [1, 8, 9]. Nevertheless, a third subtype, MT3/Mel1c, has also been identified but only found in non-mammalian species, such as birds, amphibians, and fish [10, 11]. Additionally, in mammals, a third subtype, initially identified as melatonin receptor MT3, has been further characterized as a cytosolic, non-G coupled-binding site for melatonin. It belongs to the quinone reductase family and is named quinone reductase 2 (NQO2) [12, 13]. Melatonin acts as a non-substrate inhibitor to bind to and inhibit this enzyme [14].

As members of GPCRs, activation of melatonin receptors MT1 and MT2 alters the levels of second messengers to modulate intracellular signal transduction [15]. Both MT1 and MT2 receptors inactivated adenylate cyclase (AC) and decreased intracellular cAMP production, and resulted in a decrease in protein kinase A (PKA) activity [6, 16]. Melatonin receptors also can dimerize as homo- or heterodimers to regulate cell physiological activity [17, 18]. Intriguingly, MT1 and MT2 receptors are also capable of activating very different signaling cascades in different tissues, organs or species. The MT1 receptor can increase phosphorylation of mitogen-activated protein kinase 1/2 (MAPK1/2) and extracellular signal-regulated kinase 1/2 (ERK1/2) to active the MAPK cascade. The MT2 receptor inhibits both forskolin (forsk)-induced cAMP and cGMP formation, leading to activation of protein kinase C (PKC) in the suprachiasmatic nucleus (SCN) and decrease of calcium-dependent dopamine release in the retina [19]. A growing body of evidence shows that melatonin receptors are involved in reproductive regulation [20, 21].

Leydig cells, which are located between the seminiferous tubules of the testis, are the primary cells to synthesize and secrete testosterone, an important hormone to promote the development of male reproductive tissues such as testes and prostate, as well as maintaining spermatogenesis and secondary sexual characteristics [22, 23]. Testosterone synthesis is induced by luteinizing hormone (LH) or chorionic gonadotropin (CG). Human CG (hCG) is widely used to induce testosterone synthesis [24, 25]. Testis Leydig cells, a type of endocrine secretory cells with strong testosterone synthesis and secretion in response to LH/CG stimulation, express key steroidogenic enzymes for the regulation of testosterone synthesis [24].

Treatment with LH/hCG increased intracellular levels of cAMP, and promoted the transfer of cholesterol to the inner mitochondrial membrane through steroidogenic acute regulatory protein (StAR). Then, cholesterol is converted into pregnenolone via cytochrome p450 cholesterol side chain cleavage enzymes (p450scc/CYP11A1). After movement from the mitochondria to the endoplasmic reticulum (ER), pregnenolone is converted into progesterone by 3β-hydroxysteroid dehydrogenase O5-O4-isomerase (3β-HSD) and subsequently metabolized to testosterone by 17a-hydroxylase/C17–20 lyase (CYP17) and 17β-hydroxysteroid dehydrogenase (17β-HSD) [24, 26].

In this study, we examined whether melatonin receptors are involved in regulation of hCG-induced testosterone synthesis, as well as whether melatonin receptors function via modulation of steroidogenic enzyme expression in mouse Leydig cells (mLTC-1). We further investigated the role of melatonin receptors in some cell processes including ER stress and apoptosis, attempting to find the potential signaling involved in melatonin receptor-mediated reproductive regulation.

## Materials and methods

### Animals

Male Kunming White outbred strain mice were purchased from the Laboratory Animal Center of the Fourth Military Medical University (Shaanxi, China). The mice were maintained in controlled conditions of temperature (23 ± 2 °C) and light (12 h light and 12 h dark cycle). The experimental procedures were performed in accordance with the Animal Ethical and Welfare Committee of Gansu Agricultural University.

### Cell culture

The murine Leydig tumor cell line (mLTC-1, ATCC, Manassas, VA, USA) was cultured in RPMI 1640 Medium (Hyclone, Logan, UT, USA) supplemented with 10% FBS (Hyclone) and 100 mg/L penicillin/streptomycin (Hyclone) at 37 °C under 5% CO_2_.

For testosterone assay, mLTC-1 cells were cultivated in 24-well plates (5 × 10^4^ cells/well) for 24 h before stimulation with hCG (Yofoto. Ningbo, China). Then cells were washed with phosphate-buffered saline (PBS) and maintained in alternative FBS-free/phenol red medium containing hCG with a controlled concentration gradient. After 6 h incubation, the cells and culture supernatant were collected. Cells were used for RNA and protein assay, and culture supernatant for testosterone assay.

### Immunohistochemistry (IHC)

For production of tissue sections, the testis was dissected from mice (90 days old), fixed in 4% paraformaldehyde for 24 h, dehydrated through a graded ethanol series, and embedded in paraffin. Tissue sections 7 mm thick were cut and mounted on glass slides pre-coated with poly-L-lysine solutions. Then the dehydrated sections were placed in citrate buffer (0 .1M citrate, 0.1 M sodium citrate; pH 6.0). Antigen retrieval was performed by heating in a microwave oven (750 W for 10 min twice) and cooling slowly to room temperature. Endogenous catalase deactivation was performed by immersion of slides in 0.3% (*v*/v) hydrogen peroxide in methanol for 1 h at 37 °C.

For IHC staining, the sections were washed and then incubated with 10% goat serum for 30 min at 37 °C. The sections were washed with PBS and incubated with rabbit anti-MTNR1A/1B antibody (1:200, bs-0027R/bs-0963R, Bioss, Beijing, China) overnight at 4 °C. After washing in PBS, the sections were incubated with biotinylated anti-Rabbit IgG (Sigma-Aldrich, St. Louis, USA) for 10 min at 37 °C, and then immersed in horseradish peroxidase labeled streptavidin for 10 min at 37 °C. Appropriate negative control slides were run in parallel without a primary antibody. The slides were imaged using a digital microscope (Motic, Wetzlar, Germany).

### Quantitative real-time PCR (qPCR)

Total RNA was extracted from mLTC-1 cells using TRIzol reagent (TaKaRa, Dalian, China) according to the manufacturer’s instructions. The cDNA were synthesized using a PrimeScript RT Reagent Kit (TaKaRa). Real-time quantitative PCR (qPCR) was performed using a CFX96 Touch Real-time PCR system (Bio-Rad, Hercules, CA, USA) along with the SYBR Premix Ex Taq II Kit (TaKaRa) following the manufacturer’s protocol. The sequences of the specific primers used are listed in Table 1. These reactions were repeated three times for each sample as technical replicates. Gene mRNA quantifications were performed using the 2^–∆∆Ct^ method, and the amount of transcript in each sample was normalized using Gapdh as the internal control.

**Table 1:** Primer sequences used for qPCR analyses of gene mRNAs

Gene name	GenBank Accession	Primer sequences (5′-3′)	Product length
Gapdh	NM_001289726	TCACTGCCACCCAGAAGA GACGGACACATTGGGGGTAG	185 bp
MTNR1A	NM_008639	TGTCAGCGAGCTGCTCAATG GGTACACAGACAGGATGACCA	158 bp
MTNR1B	NM_145712	GAACAGCTCAATCCCTAACTGC ACGACTACTGTAGATAGCATGGG	135 bp
p450scc	NM_001346787	AGGTCCTTCAATGAGATCCCTT TCCCTGTAAATGGGGCCATAC	137 bp
p450c17	NM_007809	GCCCAAGTCAAAGACACCTAAT GTACCCAGGCGAAGAGAATAGA	159 bp
StAR	NM_011485	TGTCAAGGAGATCAAGGTCCTG CGATAGGACCTGGTTGATGAT	334 bp
Chop	NM_007837	AGCTGGAAGCCTGGTATGAGGA AGCTAGGGACGCAGGGTCAA	134 bp
Grp78	NM_001163434	AGAAACTCCGGCGTGAGGTAGA TTTCTGGACAGGCTTCATGGTAG	176 bp
ATF4	NM_009716	CTCTTGACCACGTTGGATGAC CAACTTCACTGCCTAGCTCTAAA	226 bp
XBP1	NM_013842	TGAGTCCGCAGCAGGTG GACAGGGTCCAACTTGT	130 bp
IRE1	AF071777	GTGGTCTCCTCTCGGGTTC CCGTCCCAGGTAGACACAAAC	111 bp
p53	NM_011640	TACAAGAAGTCACAGCACAT GATAGGTCGGCGGTTCAT	267 bp
Caspase-3	NM_001284409	TGACTGGAAAGCCGAAACTC GCAAGCCATCTCCTCATCAG	101 bp
Bcl-2	NM_009741	CGAGAAGAAGGGAGAATCACAGG AATCCGTAGGAATCCCAACC	133 bp

### Immunofluorescence staining

mLTC-1 cells were cultured in a 35 mm dish with cover slips to a monolayer. Then cover slips were fixed in 4% formaldehyde for 30 min and permeabilized in 0.1% Triton X-100 for 15 min at room temperature. After being blocked with 5% BSA in PBS, cover slips were incubated with a rabbit anti-MTNR1A/1B primary antibody (1:200, Bioss) at 4 °C overnight, followed by incubation with an Alexa-Fluor 488 labeled goat anti-Rabbit IgG secondary antibody (1:300; Invitrogen) for 1 h at 37 °C. Nuclei were stained with 4,6-diamidino-2-phenylindole (DAPI, Beyotime, Beijing, China) for 5 min. Images were captured with a digital camera under a Zeiss LSM800 confocal microscope (Carl Zeiss, Germany).

### Western blotting (WB) assay

mLTC-1 cells after treatment were harvest for protein extraction using the Total Protein Extraction Kit (KeyGen, Nanjing, China). The protein concentration was determined using a BCA Protein Assay Kit (KeyGen). Equal total proteins were separated via 12% SDS-PAGE gel and electrotransferred to polyvinylidene difluoride (PVDF) membranes (Millipore, Bedford, MA, USA). The membranes were then blocked with 10% skimmed milk in TBST for 2 h at room temperature and incubated overnight at 4 °C with the following primary antibodies: anti-β-actin antibody (Sanjian Biotech, Tianjing, China), anti-MTNR1A/1B antibody (Bioss), anti-StAR antibody (CusaBio, Wuhan, China), anti-p450c17 antibody (CusaBio), anti-Grp78 antibody (CusaBio), anti-ATF4 antibody (CusaBio), and anti-phospho-IRE1 antibody (CusaBio). Then, following three washes with TBST, the membranes were incubated with the corresponding secondary antibody conjugated to horseradish peroxidase (Sigma-Aldrich) for 1 h at room temperature. Finally, bands were visualized using the Gel Imaging System (Tannon Science & Technology, Shanghai, China) and then digitized by use of ImageJ software.

### Small interfering RNA (siRNA) mediated knockdown assay

SiRNA were purchased from GenePharma (Shanghai, China). The sequences of negative control siRNA (si-NC) and si-Mtnr1a/1b are presented in Table 2. The day before transfection, ~ 5 × 10^4^ mLTC cells were seeded in 24-well plates. mLTC were transfected with 150 nM siRNA each well for 12 h using the Turbofect transfection Reagent (Thermo Fisher, Rockford, IL, USA) according to the manufacturer’s protocol for siRNA transfection. Then siRNA solution was replaced with complete culture medium and cultured for 24 h. Cells were subsequently collected for RNA and protein extraction.

**Table 2:** Sequences of small interfering RNA

Name	Sense sequences (5′-3′)	antisense sequences (5′-3′)
si-NC	UUCUCCGAACGUGUCACGUTT	ACGUGACACGUUCGGAGAATT
siMtnr1a-1	GCAUCACGGGAAUUGCCAUTT	AUGGCAAUUCCCGUGAUGCTT
siMtnr1a-2	GGUGUUCCAUUUCAUAGUUTT	AACUAUGAAAUGGAACACCTT
siMtnr1a-3	CCUCAAUGCGAUCAUAUAUTT	AUAUAUGAUCGCAUUGAGGTT
siMtnr1b-1	GGAGCUUUCUGAGCAUGUUTT	AACAUGCUCAGAAAGCUCCTT
siMtnr1b-2	CCUGAACGCCAUCAUCUAUTT	AUAGAUGAUGGCGUUCAGGTT
siMtnr1b-3	GGAACGCAGGUAACCUGUUTT	AACAGGUUACCUGCGUUCCTT

### Testosterone assays

Testosterone in culture supernatants were measured by the Testosterone ELISA kit (Beifang Biotech, Beijing, China) according to the manufacturer’s instructions. The minimum detectable concentration of testosterone was 0.05 ng/mL. The intra- and inter-assay coefficients of variation were < 10 and < 15%, respectively.

### Statistical analysis

Data were analyzed with one-way ANOVA, followed by Fisher’s least significant difference test (Fisher’s LSD) and the independent-samples Student’s t test with SPSS software (Version 20.0; SPSS, Chicago, IL. USA). *P* < 0.05 was considered significant. All data are represented as the mean ± SEM of repeated experiments (*n* = 3).

## Result

### Immunolocalization of MTNR1A and MTNR1B protein in mouse testes

**Fig. 1:**
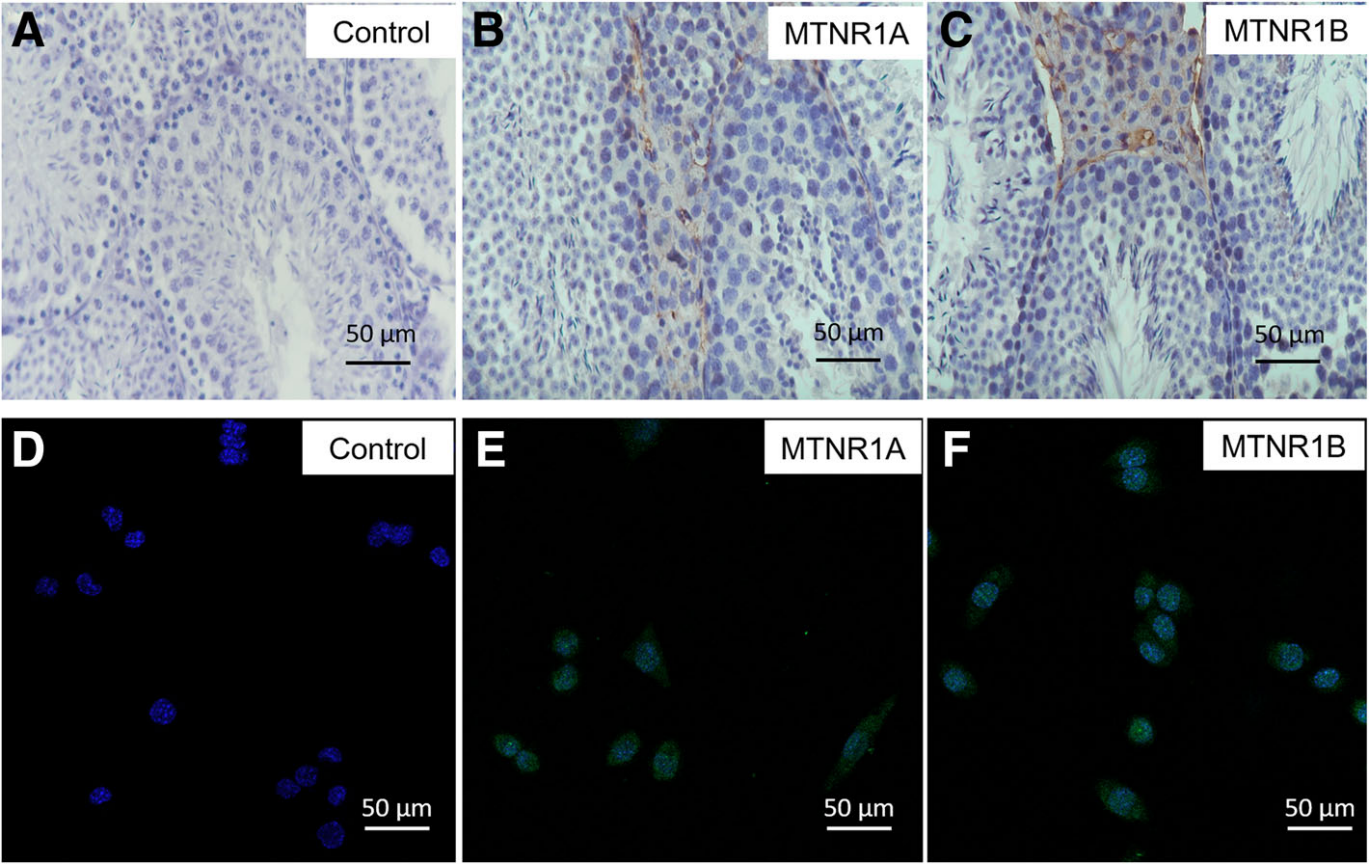
Immunolocalization of MTNR1A and MTNR1B in mouse testes and mLTC-1 cells. **a**. Control staining was performed without primary antibody but PBS. **b**. Immunohistochemistry staining showed that MTNR1A was specially labeled in Leydig cell and Sertoli cells in mouse testes. **c**. Immunohistochemistry staining showed that MTNR1B was specially labeled in Leydig cells and Sertoli cells in mouse testes. **d**-**f**. Immunofluorescence staining using anti-MTNR1A and MTNR1B antibody and Alexa-Fluor 488 labeled secondary antibody showed that both MTNR1A and MTNR1B expressed specifically on cell membrane in mLTC-1 cells. Scale bar: 50 μm

To elucidate whether MTNR1A and MTNR1B were expressed in mouse testes, we collected testis tissue during the estrous cycle and performed immunohistochemistry using antibodies against MTNR1A and MTNR1B. We found that the expression of both MTNR1A and MTNR1B was mainly distributed in Leydig cells and Sertoli cells, but not in germ cells (Fig. 1a-c), which is consistent with previous reports [10, 27].

To explore the function of melatonin receptors in vitro, we chose a mouse Leydig cell line, mLTC-1, for our research. To further confirm the expression and distribution of melatonin receptors MTNR1A and MTNR1B in mLTC-1, we performed immunofluorescence staining in mLTC-1 using specific anti-MTNR1A and MTNR1B antibody and Alexa-Fluor 488 labeled secondary antibody. We found that the melatonin receptors MTNR1A and MTNR1B are mainly located on the cell membrane (Fig. 1d-f). The result is consistent with previous reports [11, 28].

### Knockdown of melatonin receptors inhibits hCG-induced testosterone synthesis

**Fig. 2:**
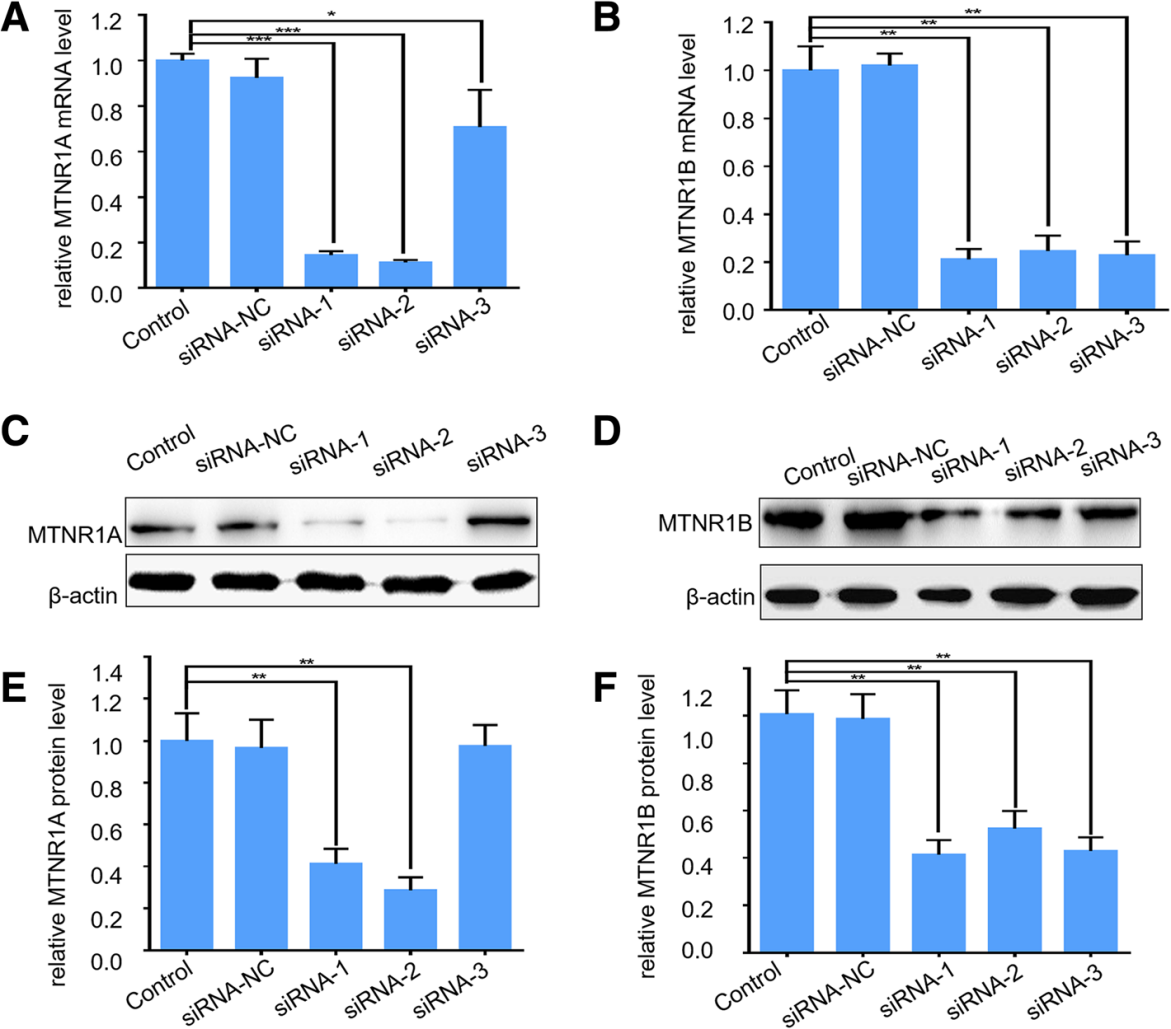
siRNA-mediated knockdown of MTNR1A and MTNR1B in mLTC-1 cells. **a** and **b**. qPCR assay of the MTNR1A and MTNR1B mRNA levels in mLTC-1 after transfection of siRNAs against MTNR1A and MTNR1B, respectively. **c** and **d**. Western blotting results of MTNR1A and MTNR1B protein levels in mLTC-1 after transfection of siRNAs against MTNR1A and MTNR1B, respectively. **e** and **f**. The intensity of WB bands in C and D was evaluated with ImageJ. Data are presented as mean ± SEM of triplicate experiments. * *P* < 0.05, ** *P* < 0.01, *** *P* < 0.001 (Student’s t-test)

To understand the biological function of melatonin receptors in mLTC-1 cells, artificial small interfering RNAs (siRNAs) against MTNR1A and MTNR1B, respectively, were used in this study. The transfection efficiency of siRNA was more than 80% in mLTC-1 (data not shown). Interference efficiency of MTNR1A and MTNR1B expression was monitored using qPCR and immunoblot. As shown in Fig. 2, compared with negative control siRNA, MTNR1A mRNA showed a significant decrease of ~ 80% after treatment with MTNR1A siRNA-1 and siRNA-2. Correspondingly, MTNR1A protein level decreased over 60% compared with the negative control (Fig. 2a, c, e). Similarly, MTNR1B was also efficiently interfered by MTNR1B siRNAs at both mRNA and protein levels (Fig. 2b, d, f). Herein MTNR1A siRNA-2 and MTNR1B siRNA-1, termed as si-Mtnr1a and si-Mtnr1b, respectively, were used for the following studies.

**Fig. 3:**
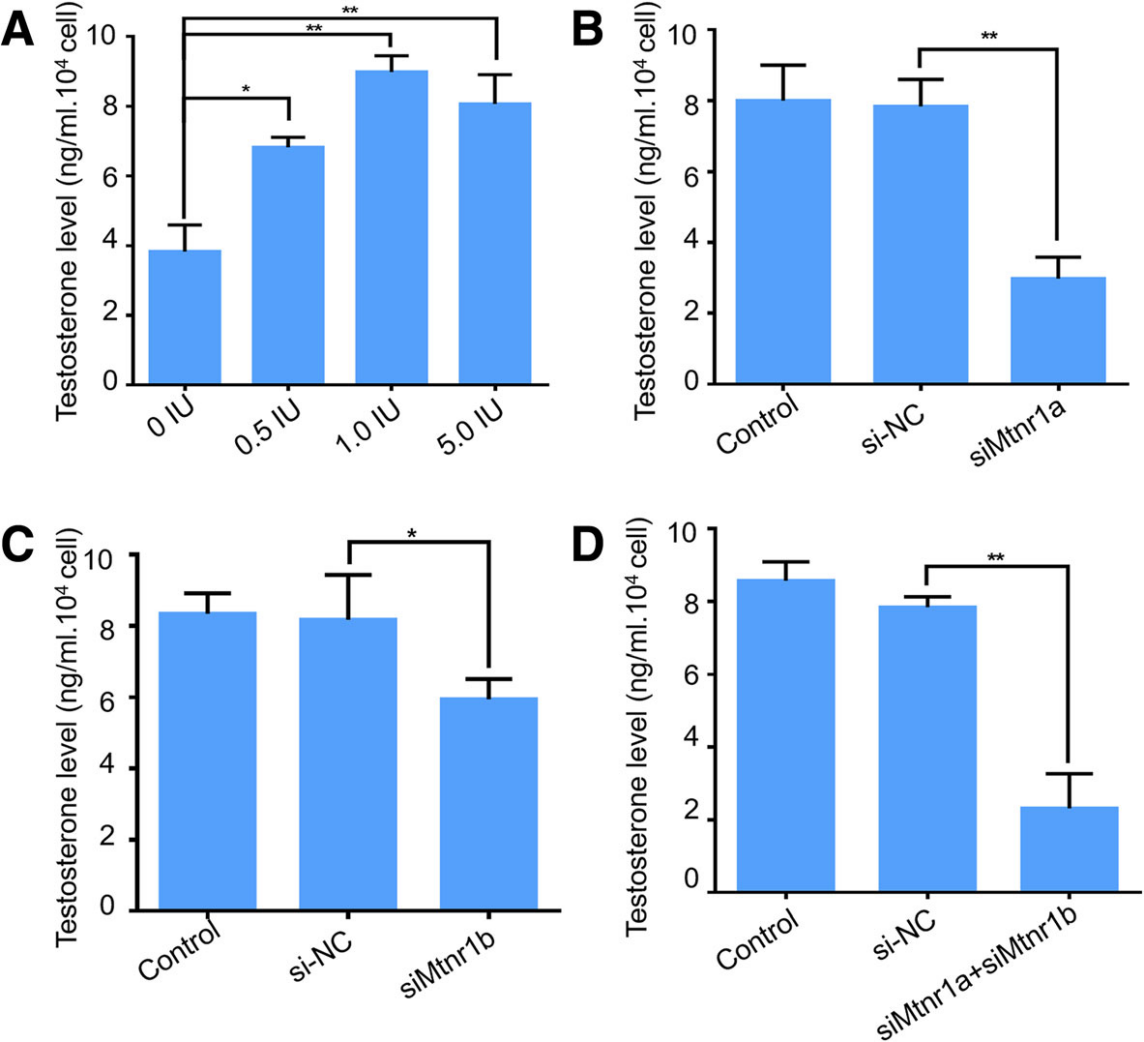
Effect of MTNR1A and MTNR1B knockdown on testosterone secretion in mLTC-1. **a**. mLTC-1 cells were treated with 0, 0.5, 1.0 and 5.0 IU/ml of hCG for 6 h before detection of testosterone level using ELISA. The results indicate that 1 IU/ml is the optimal dose for testosterone synthesis. **b** and **c**. ELISA results of testosterone secretion after interference of MTNR1A or MTNR1B using siRNA in mLTC-1. **d**. ELISA result of testosterone secretion after interference with both MTNR1A and MTNR1B in mLTC-1. Data are presented as mean ± SEM of triplicate experiments. * *P* < 0.05, ** *P* < 0.01 (Student’s t-test)

Testosterone is an important hormone to maintain male secondary sexual characteristics and spermatogenesis [22]. To understand the function of melatonin receptors in testosterone synthesis, we performed testosterone detection under the condition of siRNA-induced melatonin receptor knockdown. In this study, hCG was used to stimulate testosterone synthesis in vitro. As shown in Fig. 3, hCG stimulated testosterone synthesis in a dose-dependent manner in mLTC-1 cells, and the optimal dose is 1.0 IU/ml (Fig. 3a). Furthermore, we detected the hCG-induced testosterone secretion using ELISA after siRNA-mediated knockdown of melatonin receptors MTNR1A and/or MTNR1B in mLTC-1 cells. The results revealed that knockdown of both MTNR1A and MTNR1B inhibits testosterone synthesis (Fig. 3a, b and c). Especially MTNR1A interference led to an obvious decrease (> 60%) of testosterone level. These results suggested that melatonin receptors, especially MTNR1A, play a crucial role in hCG-induced testosterone synthesis.

### Knockdown of melatonin receptors inhibited hCG-induced steroidogenic gene expression

**Fig. 4:**
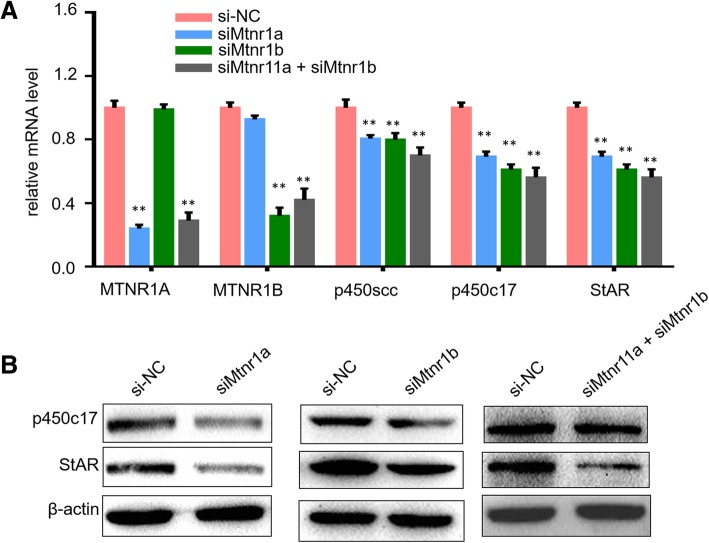
MTNR1A and MTNR1B regulated steroidogenic gene expression in mLTC-1 cells. **a**. mLTC-1 cells after transfection of si-Mtnr1a and si-Mtnr1b were treated with 1 IU/ml hCG for 6 h before monitoring of mRNA expression levels of p450scc, P450c17 and StAR using qPCR assay. **b**. Western blotting results of p450c17 and StAR protein expression in mLTC-1 after knockdown of MTNR1A and MTNR1B using specific siRNA

Steroidogenic genes play essential roles in testosterone synthesis. hCG treatment induced expression of steroidogenic enzymes [26, 29]. In order to investigate the role of melatonin receptors in hCG-induced steroidogenic gene transcription, several key steroidogenic genes including StAR, p450scc and steroid 17α-monooxygenase (p450c17) were monitored by use of qPCR and western blot after melatonin receptors’ knockdown with hCG treatment. As shown in Fig. 4, we found that knockdown of MTNR1A and MTNR1B led to an obvious decrease of StAR, P450scc and P450c17 expression at both the mRNA level and the protein level in mLTC-1 cells under hCG treatment (Fig. 4a and b). The results demonstrated that melatonin receptors including MTNR1A and MTNR1B are essential in regulation of expression of hCG-induced steroidogenic genes, such as p450scc, p450c17 and StAR, which is due to inhibition of synthesis of steroid hormones.

### Inhibition of MTNR1A and MTNR1B altered hCG-induced endoplasmic reticulum (ER) stress

**Fig. 5:**
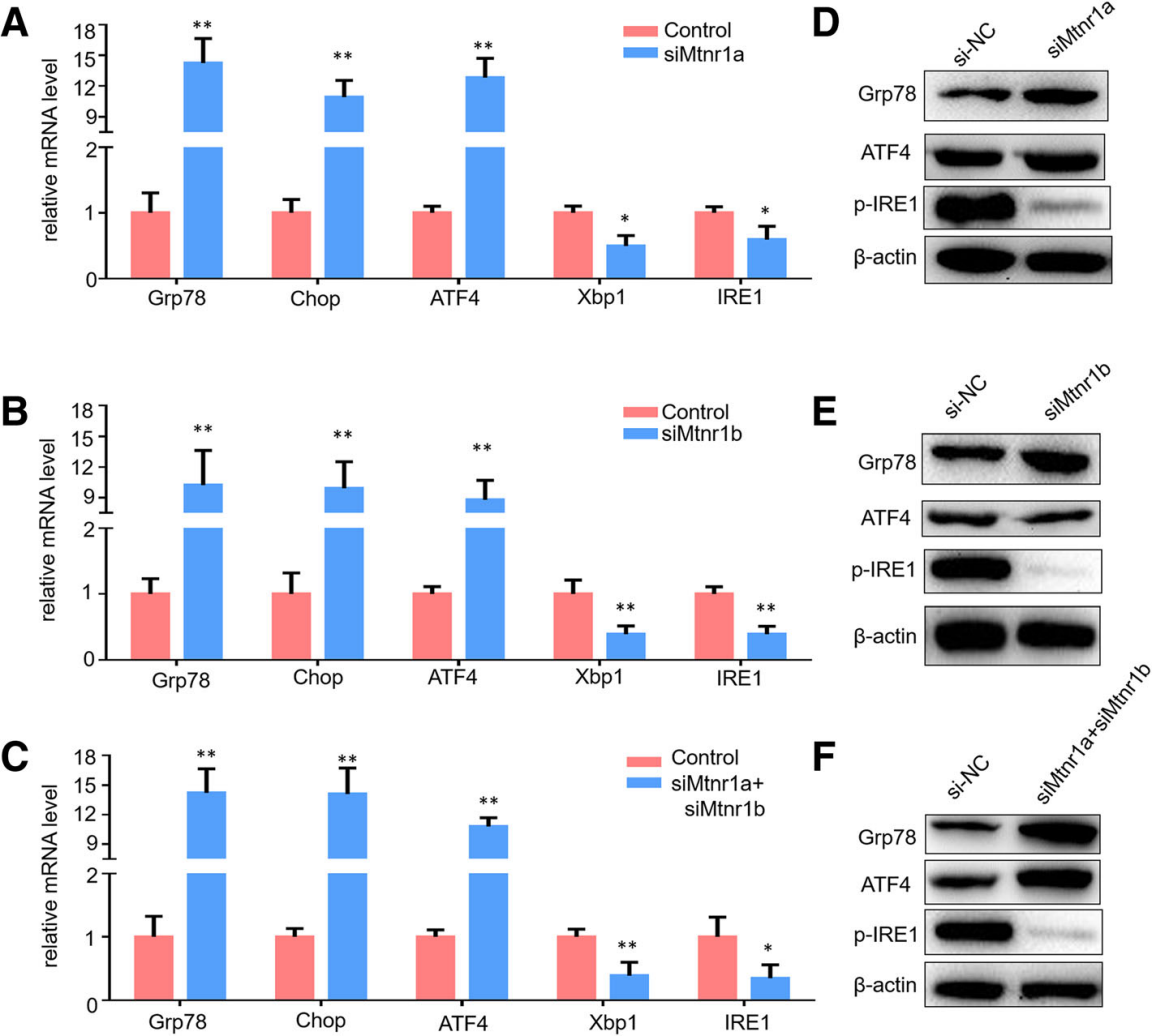
Effects of MTNR1A and MTNR1B knockdown on endoplasmic reticulum stress. **a**-**c**. Relative mRNA expression of endoplasmic reticulum (ER) stress genes, Grp78, Chop, ATF4, Xbp1 and IRE1, was evaluated using qPCR assay after treatment with the siRNAs against MTNR1A and MTNR1B for 48 h in mLTC-1. The mRNA levels were normalized to that of Gapdh. Data are presented as the mean ± SEM of three triplicates. *P < 0.05, **P < 0.01 (Student’s t-test). **d**-**f**. Western blotting analyses of Grp78, ATF4 and phospho-IRE1 expression in mLTC-1 after knockdown of MTNR1A and MTNR1B using specific siRNAs

Previous evidence indicated that hCG triggered ER stress to regulate steroidogenic gene expression [24]. To further investigate the potential roles of melatonin receptors in hCG-mediated ER stress, we examined the expression of crucial ER stress markers, including 78 kDa glucose-regulated protein (Grp78 or BiP), CCAAT/enhancer-binding homologous protein (Chop), Activating transcription factor 4 (ATF4), X-box binding protein 1 (Xbp1) and inositol-requiring enzyme 1 (IRE1) after hCG treatment (Fig. 5). First, expression of the major ER stress marker Grp78 protein dramatically increased at both mRNA and protein levels, which demonstrated that knockdown of melatonin receptors promoted hCG-induced ER stress. Three unfolded protein response (UPR) pathways are involved in ER stress regulation: Perk, ATF6 and IRE1 [30]. Notably, our study found that inhibition of MTNR1A and/or MTNR1B stimulated Chop and ATF4 expression at both mRNA and protein levels but significantly repressed Xbp1 and IRE1 expression; especially the expression of phospho-IRE1 protein, another ER stress indicator, dramatically decreased (Fig. 5a, b and c). These results suggested that MTNR1A and MTNR1B play an important role in hCG-mediated ER stress. In addition, the two melatonin receptors showed a variable response to three ER stress pathways.

### Knockdown of MTNR1A and MTNR1B promoted hCG-induced apoptosis genes

**Fig. 6:**
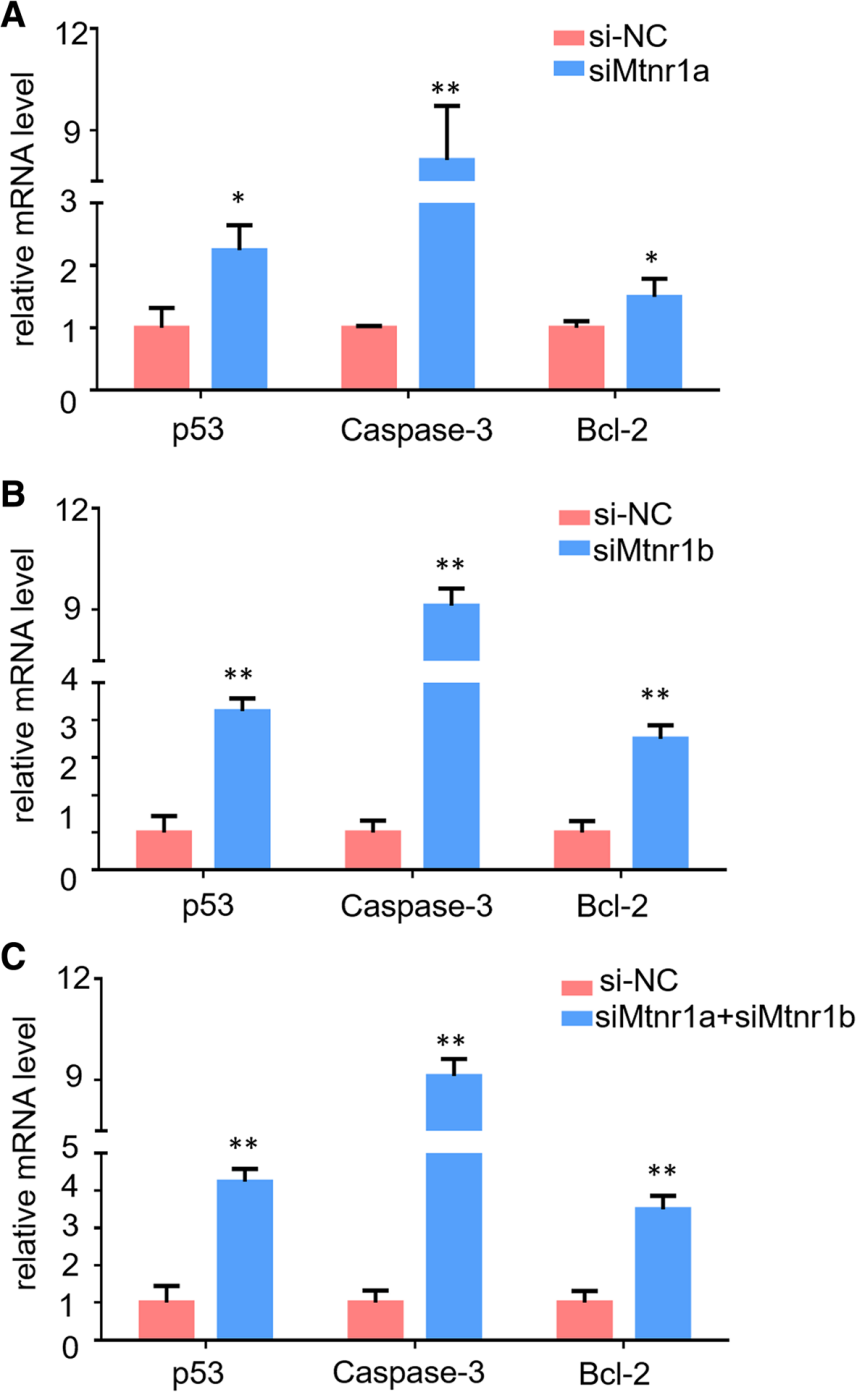
Knockdown of MTNR1A and MTNR1B promotes apoptosis genes. **a**-**c**. Relative mRNA expression of cell apoptosis marker genes, Bcl-2, p53 and caspase-3, after treatment with the siRNAs against MTNR1A and MTNR1B for 48 h in mLTC-1. The mRNA expression level was normalized to that of Gapdh. Data are presented as the mean ± SEM of three triplicates. *P < 0.05, **P < 0.01 (Student’s t-test)

Previous studies demonstrated that the hCG-induced ER stress response contributes to the induction of cell apoptosis [24, 31, 32]. When the UPR is perturbed or insufficient to deal with current stress conditions, apoptotic cell death is initiated [33]. To further explore the potential role of melatonin receptors in hCG-induced apoptosis, we collected mLTC-1 cells after knockdown of MTNR1A and MTNR1B with hCG treatment and monitored expression of several apoptosis marker genes, including p53, caspase-3 and Bcl-2 using qPCR. As shown in Fig. 6, hCG treatment significantly facilitated expression of p53 and caspase-3. Curiously, Bcl-2, an inhibitor of apoptosis [34], also was stimulated by hCG treatment after inhibition of endogenous MTNR1A and MTNR1B expression. These results strongly indicated that the melatonin receptors MTNR1A and MTNR1B play multiple roles in modulation of hCG-induced apoptosis.

## Discussion

Melatonin is an important neuro-hormone mainly synthesized by the pineal gland in mammals [2, 4]. Melatonin influences various physiological activities such as neuroendocrine function, regulation of seasonal reproduction, sexual maturation, immunoregulation, thermoregulation and some aspects of aging [1, 2]. In addition, melatonin possesses strong antioxidant activity by which it protects cells, tissues and organs from the oxidative damage caused by reactive oxygen species (ROS) [3–5].

Melatonin receptors MT1 and MT2 play crucial roles in melatonin-mediated regulation of circadian rhythms [35], the immune system [36], and control of reproduction in seasonally breeding animals [37]. Recent studies reported that melatonin facilitated spermatogenesis via MT1 and MT2 [10]. Previous evidence also revealed that hCG-induced ER stress triggered apoptosis in Leydig cells of the testis and are involved in regulation of steroidogenic genes [24], which have an important function in testosterone synthesize [26, 38]. To further explore the biological mechanisms of melatonin receptors in hCG-induced testosterone synthesis and potential cellular regulation, siRNA-mediated melatonin receptor knockdown cells, mLTC-1, were established in this study to monitor the expression of related factors.

Melatonin receptors, including MT1 and MT2, are highly expressed in mouse Leydig cells both in tissue and the cell line (Fig. 1), and expectably, siRNA-mediated knockdown of melatonin receptors significantly inhibited hCG-induced testosterone synthesis (Fig. 3) and suppressed expression of steroidogenic genes, such as p450scc, p450c17 and StAR (Fig. 4). These results clearly demonstrated that melatonin receptors, including MT1 and MT2, play essential roles in hCG-induced steroid hormones synthesis.

The ER plays a crucial role in the synthesis and folding of secretory and membrane proteins in eukaryotic cells. Overload of ER functions, including excessive protein synthesis, Ca^2+^ homeostasis, and accumulation of unfolded and/or misfolded proteins in the ER lumen, lead to ER stress through activation of the UPR through three ER transmembrane proteins-mediated signaling: Perk, ATF6 and IRE1 [30]. Previous evidence has confirmed that hCG-induced ER stress plays an important role in steroidogenic enzyme regulation through the activation of UPR pathways [24]. In our studies, we found that knockdown of melatonin receptors obviously promoted hCG-induced major ER stress marker Grp78 protein expression (Fig. 5) and increased Chop and ATF4 expression, indicating that melatonin receptors play crucial roles in inhibiting hCG-induced ER stress. Interestingly, the expression of phospho-IRE1 protein, another ER stress indicator, dramatically decreased after knockdown of melatonin receptors. Together with the result that the expression of IRE1 and Xbp1 (a downstream functional transcriptional activator of IRE1) mRNA was also repressed significantly, these results demonstrated that hCG may modulate IRE1 signaling pathways in a melatonin receptor-dependent manner.

Although the UPR mediates physiological regulation or homeostasis of the ER, it can also mediate apoptotic signaling pathways under excessive ER stress [31, 32, 39, 40] through the activation of caspase-12 [33, 41]. In our study, we tested expression of some apoptosis markers after knockdown of melatonin receptors under hCG treatment. Expectably, inhibition of MTNR1A and MTNR1B promoted expression of key apoptosis markers, including p53 and caspase-3 (Fig. 6), which may contribute to the activation of UPR pathways after hCG stimulation. Curiously, Bcl-2, which is localized to the outer membrane of mitochondria and plays an important role in promoting cellular survival and inhibiting the actions of pro-apoptotic proteins [34], is also stimulated after knockdown of melatonin receptors under hCG treatment. These findings demonstrated that knockdown of melatonin receptors under hCG treatment can cause complex changes in cellular signaling pathways. The underlying mechanisms need to be further elucidated.

In conclusion, our study demonstrated that mammalian melatonin receptors MT1 and MT2 are involved in testosterone synthesis. Knockdown of melatonin receptors inhibits hCG-induced testosterone synthesis via inhibiting steroidogenic gene expression. In addition, melatonin receptors play variable roles in regulation of three ER stress pathways. In general, the melatonin receptors MTNR1A and MTNR1B are essential to repress hCG-induced ER stress and cell apoptosis, which indicated that melatonin receptors play a crucial role in maintaining homeostasis in Leydig cells. Notably, hCG may modulate IRE1 signaling pathways in a melatonin receptor-dependent manner. We will attempt to identify the underlying mechanisms of melatonin receptor-mediated regulation of ER stress and apoptosis pathways in a further study.

## Abbreviations

3β-HSD: 3β-hydroxysteroid dehydrogenase O5-O4-isomerase
ATF4: Activating transcription factor 4
Chop: CCAAT/enhancer-binding homologous protein
ELISA: Enzyme-linked immunosorbent assay
ER: Endoplasmic reticulum
Grp78/BiP: 78 kDa glucose-regulated protein
hCG: human chorionic gonadotropin
IHC: Immunohistochemistry
IRE1: Inositol-requiring enzyme 1
LH: Luteinizing hormone
MAPK: Mitogen-activated protein kinase
mLTC-1: murine Leydig tumor cell line
MT/MTNR: Melatonin receptors
p450c17: steroid 17α-monooxygenase
p450scc/CYP11A1: cytochrome p450 cholesterol side chain cleavage enzymes
PKC: Protein kinase C
qPCR: quantitative real-time PCR
siRNA: small interfering RNA
StAR: Steroidogenic acute regulatory protein
UPR: Unfolded protein response
Xbp1: X-box binding protein 1
